# The Effect of Statins on the Incidence and Prognosis of Bladder Cancer: A Systematic Review and Meta-Analysis

**DOI:** 10.3390/curroncol30070488

**Published:** 2023-07-12

**Authors:** Panagiotis Symvoulidis, Constantinos Tsioutis, Constantinos Zamboglou, Aris P. Agouridis

**Affiliations:** 1 School of Medicine, European University Cyprus, Nicosia 2404, Cyprus; ps172284@students.euc.ac.cy (P.S.); k.tsioutis@euc.ac.cy (C.T.); constantinos.zamboglou@goc.com.cy (C.Z.); 2 Department of Radiation Oncology, Medical Center University of Freiburg, 79106 Freiburg, Germany; 3 Department of Internal Medicine, German Oncology Center, Limassol 4108, Cyprus

**Keywords:** statins, HMG-CoA reductase inhibitors, urinary bladder cancer, systematic review, meta-analysis

## Abstract

Background: Statins are widely used due to their ability to lower plasma cholesterol and offer protection from the effects of atherosclerosis. However, their role in urology and specifically bladder cancer remains unclear. We aimed to systematically address this issue in the literature and determine any possible effects of statin therapy on bladder cancer. Methods: We searched MEDLINE (PubMed) and Cochrane Library databases for records up to 26 March 2023, for studies evaluating the effects of statins on urinary bladder cancer (UBC). We included all randomized controlled trials (RCTs), cohorts, and case-control studies that were conducted on the adult population. PROSPERO registration number: CRD42023407795. Results: Database searches returned 2251 reports, and after thorough investigation and assessment for eligibility, 32 reports were included in the analysis. Of them, 4 were RCTs, 6 were case-control studies, and 22 were cohort studies. Our qualitative analysis demonstrated no association between statin administration and UBC local control, recurrence, survival, or mortality, or between statin administration and bacille Calmette–Guérin (BCG) immunotherapy effectiveness. A meta-analysis of 10 trials revealed a non-significant reduction of 11% in UBC risk among users compared with non-users in RCTs (RR: 0.89, 95% CI 0.68–1.16, *p* = 0.37) and a non-significant increase of 32% of UBC risk among statin users compared with non-users in the analysis of the cohort studies (RR: 1.32, 95% CI 0.76–2.30, *p* = 0.33). Conclusions: Our results provide strong evidence to support the neutral effect of statins on UBC local control, recurrence, survival, and mortality, and on BCG immunotherapy. Our meta-analysis revealed a non-significant effect on UBC risk among statin users when compared with non-users, indicating no statin effect on UBC incidence and overall prognosis.

## 1. Introduction

Urinary bladder cancer (UBC) is the 10th most commonly diagnosed cancer worldwide [1]. Smoking is the most important risk factor, accounting for almost half of all cases. Additional risk factors include occupational exposure to aromatic amines, polycyclic aromatic hydrocarbons and chlorinated hydrocarbons, and exposure to arsenic in drinking water and chlorination of drinking water [1]. Commonly, when referring to UBC, the specific pathologic subtype is that of urothelial carcinoma, unless otherwise specified. Additionally, UBC is generally classified as non-muscle invasive bladder cancer (NMIBC), muscle-invasive bladder cancer (MIBC), or advanced bladder cancer for higher stages [1]. NMIBC involves flat tumors confined to the mucosa known as carcinoma in situ (CIS or Tis), and papillary tumors confined to the mucosa (Ta) or invading the lamina propria (T1). NMIBC is subcategorized based on stage, grade, and other pathological features.

Statins, also known as 3-hydroxy-3-methylglutaryl coenzyme-A reductase (HMG-CoA reductase) inhibitors, are commonly prescribed for either primary or secondary prevention of atherosclerotic cardiovascular disease (ASCVD) [2]. The available statins on the market include simvastatin, lovastatin, atorvastatin, fluvastatin, pravastatin, rosuvastatin, and pitavastatin. The most common side effects of statins are myopathy/myalgia, rhabdomyolysis, and hepatotoxicity [3,4]. A diabetogenic effect of statins has also been reported [5]. Usually symptoms are dose-dependent and well-tolerated without the need for intensive monitoring with lab tests. However, despite being well-documented and their risk for cancer being relatively disproven with meta-analyses of RCTs [6], their association with UBC has not been exclusively investigated.

To this end, attempts have been made to find an association between UBC and statins. So far, several studies have reported benefits, while other studies have found an increased UBC risk in patients treated with statins, but the existence of any relationship remains controversial. Moreover, several reports suggest statins’ pleiotropic or anti-inflammatory effects could be beneficial in cancer treatment [7]. Potential pathways that could explain statins’ beneficial effects on UBC include reduction in neovascular formation, cell proliferation, inhibition of selenoprotein synthesis, and decreased natural killer cell function [6]. Considering the widespread use of statins, as well as the increasing average age and the high incidence of cancer, the identification of any relationship between statins and UBC could be significant for public health.

The current study aims to conduct a systematic review of all available evidence in the literature, including randomized controlled trials (RCTs), case-control trials, and cohorts, examining any association between current statin therapy, specifically with atorvastatin, fluvastatin, lovastatin, pitavastatin, pravastatin, rosuvastatin, and simvastatin, on the incidence and prognosis of UBC. Specifically, it aims to examine the presence of any association between statins and NMIBC incidence, local control, local disease recurrence, progression to surgery (namely cystectomy), survival, mortality, and bacille Calmette–Guérin (BCG) immunotherapy effectiveness.

## 2. Materials and Methods

### 2.1. Study Design

We performed a qualitative synthesis of RCTs and case-control and cohort studies to determine any effects of statin therapy, specifically of atorvastatin, fluvastatin, lovastatin, pitavastatin, pravastatin, rosuvastatin, and simvastatin, on bladder cancer.

### 2.2. Search Strategy

This systematic review was registered on PROSPERO (ID number: CRD42023407795) and was conducted in accordance with the Preferred Reporting Items for Systematic Reviews and Meta-Analyses (PRISMA) guidelines [8]. An extensive search of the MEDLINE and Cochrane Library databases was conducted up to the 26th of March 2023 for eligible studies with no date limit, using combinations of the following keywords: (“statin”, “atorvastatin”, “fluvastatin”, “lovastatin”, “pitavastatin”, “pravastatin”, “rosuvastatin”, “simvastatin”, and “HMG-CoA reductase inhibitors”) and (“bladder cancer”, “transitional cell carcinoma”, and “urothelial carcinoma”). Medical subject heading (MeSH) terms were also used to broaden and ensure the completeness of our search by including indexing for similar possibly unaccounted keywords. Additionally, the references of all eligible studies were manually searched for any relevant studies that might have eluded our initial search and meet the PRISMA criteria. This search only included papers written in English.

### 2.3. Eligibility Criteria

Eligible studies for our systematic review were RCTs, case-control studies, and cohort studies in adults (≥18 years old) that compared statin therapy (atorvastatin, fluvastatin, lovastatin, pitavastatin, pravastatin, rosuvastatin, and simvastatin) with placebos or their equivalent in a control group. We excluded any studies focusing on pediatric populations, articles with insufficient or incomplete data, and articles not written in English. Eligibility criteria were formed based on the PICOS (population, intervention, comparator/controls, outcomes, and study design) study question format and are summarized in Supplementary Table S1.

### 2.4. Data Extraction

Two authors (P.S., A.P.A.) independently scanned the title and abstract of every record and assessed the full text to determine studies that met all the inclusion criteria to be included in the review. During this process, any disagreement was resolved by means of a consensus-seeking discussion between the aforementioned authors (P.S., A.P.A.). Data extraction was performed following the PRISMA model. Because of the study’s design, there was no need for approval by the National Bioethics Committee (CNBC) or for informed permission from the patients. Following the initial database query, all results were screened for duplicates using the Zotero (version 6.0.26) software. Next, the following data were extracted: first author, publication year, study type, country where the trial was conducted, number and characteristics of the participating population, and the outcome. Finally, papers were chosen based on a careful full-text review for the final selection to be made.

### 2.5. Assessment of Bias

A risk of bias assessment was performed for each included study to establish transparency of systematic review results and findings. For the assessment of eligible RCTs, the revised Cochrane risk-of-bias tool for randomized trials (RoB-2) [9] was used. Based on this algorithm, studies are classified as ‘low-risk’, ‘high-risk’ or ‘with some concerns’ regarding bias. Five distinct domains were evaluated for bias arising from the randomization process, bias due to deviations from intended interventions, bias due to missing outcome data, bias in the measurement of the outcome, and bias in the selection of reported results. Traffic light and summary figures were generated using the robvis tool [10].

The Newcastle–Ottawa scale (NOS) was used for the assessment of case-control studies and cohorts [11]. NOS addresses three main domains regarding bias: selection, comparability, and exposure. In each domain there exist subcategories allowing for in-depth assessment. On each category, stars can be awarded for meeting certain quality standards. In selection, comparability, and exposure, up to four (4), two (2), and three (3) stars can be awarded, respectively, for a maximum of nine (9). Depending on the total score, a study is characterized as being of high quality (score 7–9), high risk for bias (score 4–6), or very high risk for bias (score 0–3).

Publication bias was evaluated using funnel plots of effect estimates against their standard errors (on a reversed scale). Funnel plots were created using Review Manager 5 (RevMan 5, version 5.4, Copenhagen: The Cochrane Collaboration, 2020). Asymmetry in the funnel plot indicates publication bias.

### 2.6. Quantitative Analysis

Following careful examination of the reports, a meta-analysis of selected RCTs and cohorts was performed. Pooled estimations regarding the outcomes were expressed as dichotomous for UBC. Meta-analyses were performed using a random effects model or a fixed effects model, as deemed appropriate. For dichotomous data, pooled risk-ratios (RR) and 95% confidence intervals (CIs) were calculated. A statistical analysis was performed, and forest plots were generated using Review Manager 5 (RevMan 5, version 5.4, Copenhagen: The Cochrane Collaboration, 2020). A *p*-value of less than 0.05 was considered significant. Heterogeneity was assessed using the I-squared (I^2^) test. The heterogeneity was considered as low, moderate, or high if the I^2^ was 25%, 50%, or higher than 75%, respectively. If the *p*-value was less than 0.10, the random effects model was adopted; otherwise, the fixed effects model was used.

## 3. Results

### 3.1. Study Selection

**Figure 1:**
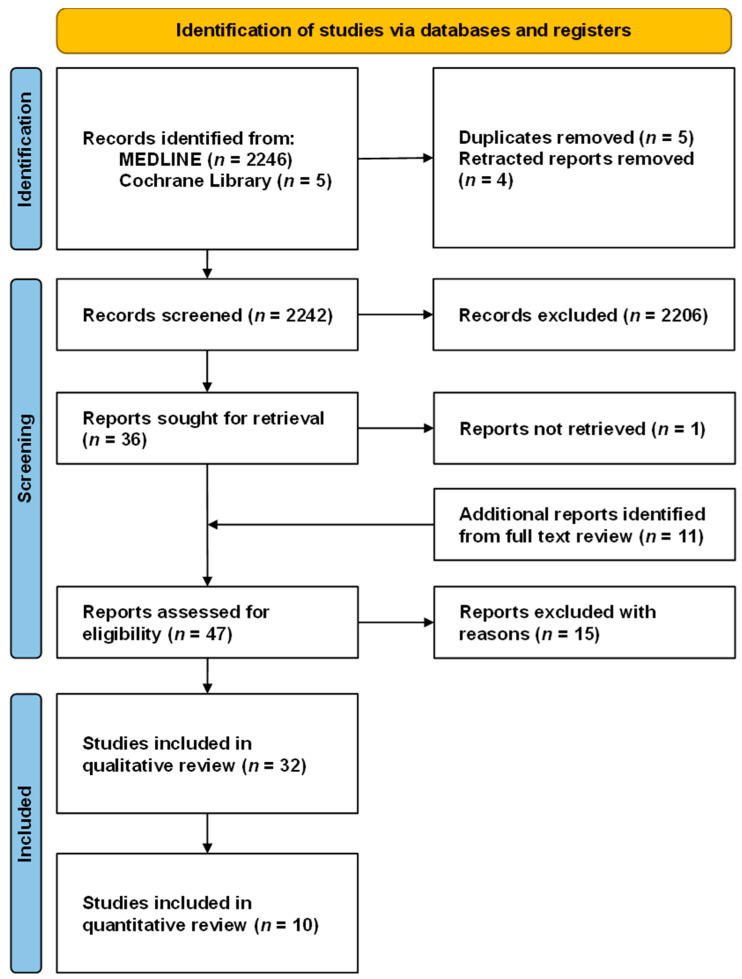
Prisma flowchart of our systematic review.

The selection process is reflected on the PRISMA flowchart, seen in Figure 1. The initial search returned a total of 2251 results. Retracted and duplicate reports were detected with automation software and removed from the screening process. Titles and abstracts were screened for relevance. After the initial screening, full-text articles for the remaining entries (*n* = 47) were assessed for eligibility. Systematic reviews and meta-analyses were reviewed for possible missed articles but excluded from the quantitative analysis. In the end, after the exclusion of ineligible studies (see Supplementary Table S2) [12,13,14,15,16,17,18,19,20,21,22,23,24,25,26], a total of 32 studies were judged as eligible for our qualitative review, of which 10 were included in the meta-analysis.

### 3.2. Study Characteristics

The characteristics of the included studies can be seen in Table 1. Our search yielded 32 reports as eligible results. Of them, 4 were RCTs, 6 were case-control studies and 22 were cohort reports. Reports that examined and reported UBC incidence included four RCTs [27,28,29,30], five case-control studies [31,32,33,34,35], and seven cohort [36,37,38,39,40,41,42] studies. Eight cohort [14,43,44,45,46,47,48] studies reported information on local control and recurrence. Progression to further surgery, specifically cystectomy, was examined in four cohort [44,45,49,50] studies. Two studies provided information regarding survival, and six studies [44,45,49,50,51,52] investigated the possible effects of statins on BCG immunotherapy. One RCT investigated lovastatin, while the rest investigated simvastatin. The vast majority of studies did not distinguish between individual statins when reporting data.

### 3.3. Outcomes of the Included Studies

The outcomes of interest in the included studies are described in Table 1. In brief, our qualitative analysis demonstrated no association between statins’ administration and UBC local control, recurrence, survival, or mortality, or between statins’ administration and the effectiveness of BCG immunotherapy.

### 3.4. Results from the Quantitative Synthesis

**Figure 2:**
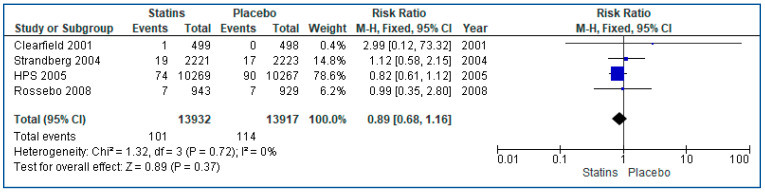
Forest plot of statins vs. placebos in RCTs, outcome: statins on bladder cancer risk [27,28,29,30].

**Figure 3:**
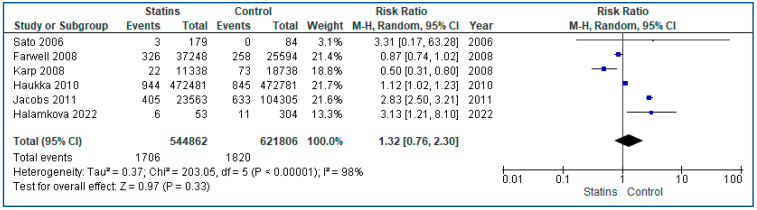
Forest plot of statins vs. controls in cohort studies, outcome: statins on bladder cancer risk [36,37,39,40,41,42].

Regarding the UBC risk on statin patients, we performed a meta-analysis of four RCTs [27,28,29,30] and six cohort [36,37,39,40,41,42] studies (Supplementary Table S3). The included population consisted of 13,932 statin users and 13,917 controls for the RCTs analysis, and 544,862 users and 621,806 controls for the cohort studies. Regarding RCTs, we used a fixed-effects model for the analysis and found a non-significant reduction of 11% in UBC risk among users compared with non-users (RR: 0.89, 95% CI 0.68–1.16, *p* = 0.37). In contrast, because of very high heterogeneity (I^2^ = 98%), we used a random-effects model for the analysis of the cohort studies. Pooled analysis showed a non-significant increase of 32% in UBC risk among users compared with non-users in the analysis of the cohort studies (RR: 1.32, 95% CI 0.76–2.30, *p* = 0.33). Forest plots of comparisons for RCTs and cohorts can be seen in Figure 2 and Figure 3, respectively.

### 3.5. Publication Bias

**Figure 4:**
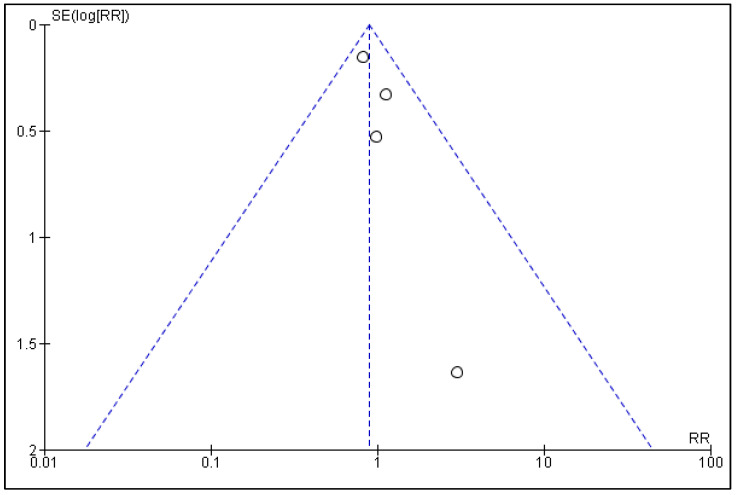
Funnel plot of statins vs. placebos in randomized controlled trials evaluating the presence of publication bias.

**Figure 5:**
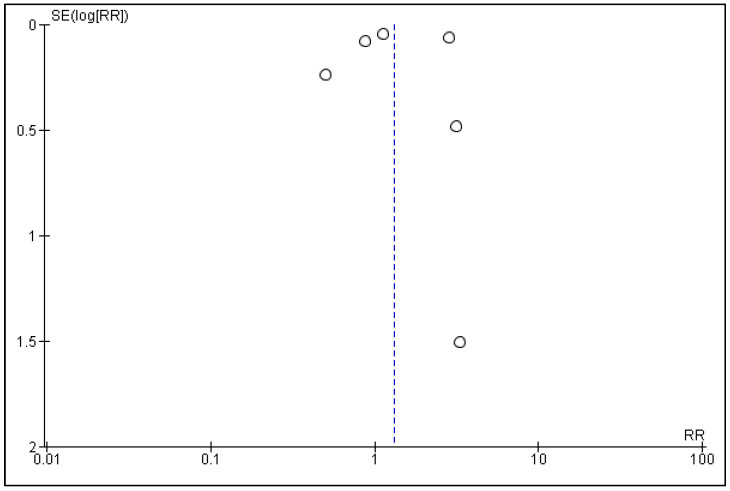
Funnel plot of statins vs. controls in cohort studies evaluating the presence of publication bias.

Funnel plots for the comparison of statins vs. placebos/controls in RCTs and cohort studies can be seen in Figure 4 and Figure 5, respectively. Publication bias for the current meta-analysis was difficult to estimate because only four RCTs and six cohorts were included. However, the funnel plot for RCTs appeared to be symmetrical on visual inspection, indicating no publication bias, while asymmetry was noted in the funnel plot of cohort studies, indicating publication bias.

### 3.6. Quality Appraisal

**Figure 6:**
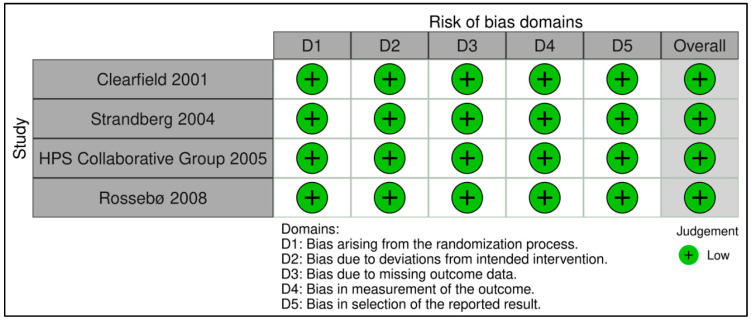
Traffic light plot displaying risk of bias assessment according to the authors’ opinion of included RCTs on each domain of risk as described in the RoB2 tool [27,28,29,30].

**Figure 7:**
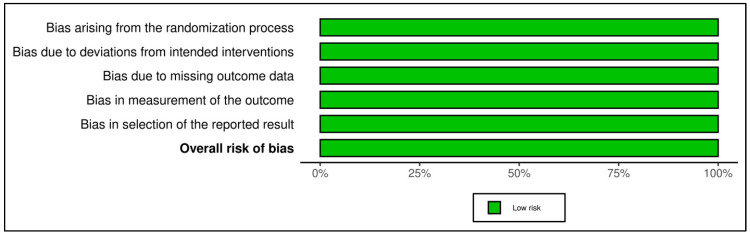
Summary plot displaying risk of bias assessment according to the authors’ opinion of included RCTs on each domain of risk as described in the RoB2 tool.

The included studies were appraised according to the methods defined in the review protocol. RCTs were appraised using the RoB2 tool (Figure 6 and Figure 7); case-control studies (Table 2) and cohort studies (Table 3) were assessed using the NOS. All four RCTs were judged to have a low risk of bias. The mean value for the six case-control studies included in this review was 7 points indicating a moderate risk of bias, while the mean value for cohorts was 7.82 points indicating that the included studies are of high quality.

## 4. Discussion

### 4.1. Incidence

UBC incidence was the primary outcome of this systematic review and meta-analysis. Reports that examined and reported UBC incidence included four RCTs [27,28,29,30], five case-control studies [31,32,33,34,35], and seven cohort studies [36,37,38,39,40,41,42].

All RCTs reported similar rates, and as seen in the meta-analysis, the observed reduced risk was not significant (*p* = 0.37). It might be worth noting that one reported trial [27] only provided information for women, and as such, the data could be considered incomplete as a significant part of the original population is missing.

Case-control studies reported overall similar rates with the exception of two studies that reported non-significant results. Considering that the included case-control studies were identical to those of the 2013 meta-analysis by Zhang et al. [58], we elected not to perform another meta-analysis using identical data.

In our analysis of cohorts, we found a non-significant increase in bladder cancer risk among users compared with non-users (*p* = 0.33). The observed increased risk in the study by Sato et al. [36] was determined from comparison with the expected reference population rates (O/E: 13.76, 95% CI 2.77–40.21). In the study by Friedman et al. [38], there was an increased risk for bladder cancer in men and women that remained significant even after adjustment for smoking, but adjusted rates are not reported. Karp et al. [39] studied statins on ‘high/low-dose’ and ‘no-use’ groups (‘high-dose’ HR: 1.1, 95% CI 0.4–1.8, ‘low-dose’ HR: 1.3, 95% CI 0.6–2.0, ‘no-use’ HR: 2.4, 95% CI 1.8–2.9). The study concluded that the existence of a dose–response relationship between statin dose and cancer incidence was possible, but the increased risk was not observed for bladder cancer specifically. Haukka et al. [40] found no change in risk between statin users and non-users (RR: 1.08, 95% CI 0.99–1.19) and a similar incidence rate per year of exposure to any statin (RR: 1.01, 95% CI 0.97–1.05) but were again unable to adjust for important factors including age, sex, and smoking. Jacobs et al. [41] investigated treatment duration and incidence and found no change in risk for former users (RR: 0.87, 95% CI 0.63–1.19), short-term users (RR: 1.10, 95% CI 0.93–1.31), or long-term users (RR: 0.98, 95% CI 0.81–1.20), and these were reported to be not statistically significant (*p*-values not reported). The increased risk reported in the study by Farwell et al. [37] was not statistically significant (univariate HR: 0.94, 95% CI 0.80–1.11, multivariate HR: 0.94, 95% CI 0.77–1.13, *p*-trend = 0.25), and in the study by Halámková et al. [42], although the percentage of patients with UBC was higher on the user group (3.6% vs. 11.3%, or 11/304 vs. 6/53, *p* = 0.015, compared to a population reference of 2.9%), important factors such as smoking and obesity were not addressed.

Taking all the conclusions and limitations of the cohort studies into consideration, a neutral effect of statins overall was expected from the quantitative analysis. It is worth observing that the analysis of the RCTs revealed an 11% reduction, while the cohorts revealed a 32% increase in risk in the user group compared with the non-user group (*p* = 0.37 and *p* = 0.33 for the comparisons, respectively). Our results seem to concur with those of the preceding 2013 meta-analysis by Zhang et al. [58] that found no association between statin use and the risk of UBC.

### 4.2. Local Control and Recurrence

Regarding local control and absolute disease recurrence, current evidence suggests no effect of statins. Eight cohort studies [14,43,44,45,46,47,48,50] reported information on local control and recurrence. Three studies [14,47,48] found increased rates, while two studies [43,50] found reduced rates in users compared with non-users. The remaining three studies found similar rates in both groups.

Among the reports that found positive associations, da Silva et al. [14] found an association between statin use and recurrence on univariate analysis, and Ferro et al. [48] found increased rates of residual tumor tissue at re-TURB. Pastore et al. [47] also found an increased recurrence number among statin users compared with non-users on univariate analysis. In the reports that found reduced rates among statin users compared to non-users, Tsai et al. [43] only observed this association in univariate analysis, and in the report by Singla et al. [50] a similar association was also observed but with no significance.

Interestingly, all of the above studies found these associations in univariate analyses without being able to establish statin use as an independent risk factor in multivariate analysis. Despite these hints, it remains uncertain whether statins have any impact on local control or absolute recurrence. Additionally, regarding RFS, the included studies concluded that there is no association, which might also further support the absence of an association. Based on this evidence, we conclude that statin therapy has no effect on local control and recurrence.

### 4.3. Progression to Cystectomy

Current evidence suggests that statin users compared to non-users show similar rates of progression to cystectomy. The need for progression to further surgery, specifically cystectomy, was examined in four cohort [44,45,49,50] studies. The first study by Hoffmann [49], which reported higher rates in statin users, was a small cohort of patients with a disproportionate sample of cases compared to controls. The study by Singla et al. [50] found this association to be not statistically significant (HR 1.40, 95% CI 0.58–3.37, *p* = 0.449). There have been no large RCTs or cohort studies examining statin use as a chemopreventive agent in UBC, and as such, it is difficult to draw any conclusions. That said, considering current evidence pointing towards a neutral effect of statins in relation to local control and absolute recurrence, it could logically be inferred that statins would not have any effect on disease progression and the need for cystectomy.

### 4.4. Survival and Mortality

Two studies provided information regarding survival in this review. The study by Richard et al. [54] reported a cumulative improvement in OS per year in patients taking statins following NMIBC diagnosis, while the study by Haimerl et al. [57] did not find any association with RFS, CSS, or OS. Regarding the first study, no such improvement was observed in patients that took statins before the diagnosis (HR 1.01, 95% CI 0.99–1.03, *p* = 0.10). Considering the results of the second study as well as the author’s comments on this finding, the improvement in OS could be attributed to other factors, namely, the well-established cardiovascular effects of statins, which have also previously been the topic of great attention in studies illustrating selection bias and immortal-time bias [59].

Six cohort studies [14,44,46,48,50,51] reported information on mortality. All studies reported similar rates between the groups. In a study by Skolarus et al. [51], the difference in mortality rates (25.6% vs. 34.0%, *p* = 0.38) was attributed to chance. In another study by da Silva et al. [14], an association was found on univariate analysis (HR 1.26, 95% CI 1.04–1.54, *p* = 0.02) but not upon multivariate analysis (HR: 1.04, 95% CI 0.84–1.28, *p* = 0.68), and similarly, in the study by Singla et al. [50], an improvement in cancer-specific mortality was not significant (HR: 0.27, 95% CI 0.05–1.49, *p* = 0.133).

Overall, in regard to survival and mortality, current evidence does not suggest the presence of an association.

### 4.5. Bacille Calmette–Guérin Immunotherapy

A 2006 retrospective cohort by Hoffmann P [49] of 84 patients receiving BCG immunotherapy for NMIBC first observed more aggressive tumor behavior in patients taking statins compared to those who were not (53% vs. 18%, OR: 4.9, 95% CI 1.64–14.69, *p* = 0.004), and a greater number progressed to cystectomy (42% vs. 14%, OR: 4.5, 95% CI, 1.43–14.30, *p* = 0.01). The number of metastases was similar between the groups. These findings led to a suggested benefit from statin discontinuation during BCG immunotherapy. This sparked a series of prompt studies [44,45,50,51,52] that failed to confirm these associations. Additionally, a 2021 systematic review and meta-analysis by Cai et al. [60] examined the effects of statins and fibrin clot inhibitors (specifically, aspirin, clopidogrel, and warfarin) on BCG and also concluded that these agents do not affect BCG therapy or prognosis. Our results also support this conclusion, as we did not observe any effect of statin therapy on BCG performance among either group.

### 4.6. Role of Statins in Bladder Cancer

Currently, statin therapy in UBC is thought of as not having any direct or recognizable benefits. The theoretical background relying on statins’ well-documented pleiotropic effects exists and supports potential benefits based on metabolism control and gene regulation. To date, statins have shown limited efficacy in monotherapy trials, which hints that their subtle antitumor effects might be better appreciated in combination therapies.

Cholesterol and lipid metabolism have been linked with cellular transformation [61] and the release of inflammatory cytokines [62]. An example is the peroxisome proliferator-activated receptor gamma (PPARγ) that belongs to a family of nuclear hormone receptors and regulates lipid metabolism as a sensor. The activation of PPARγ has been shown to exert anti-inflammatory and antineoplastic effects through the inhibition of proliferation or cell migration [63]. It has been observed that patients with higher activation of PPARγ show better survival rates [64], but its role in UBC remains controversial. Preclinical studies have demonstrated statins’ regulation of this pathway with desired benefits [64,65], such as tumor cell apoptosis and the inhibition of proliferation.

Another incentive for investigating statins as potential adjuncts in cancer treatment is their antiproliferative and apoptotic effects and synergistic action when combined with other therapeutics in order to overcome resistance, demonstrated in preclinical and in vitro studies [66]. However, despite the possibility of drug repurposing being an attractive opportunity, it must not be overlooked that previous reports, as well as this study, have not been able to observe any beneficial effects of statins in UBC therapy. We conclude statins have no role in UBC therapy in the context of current clinical practice, but considering the supporting theoretical background and the complexity of cancer metabolism, the possibility of a beneficial use as an adjunct in anticancer regimens cannot be ruled out.

### 4.7. Limitations

Our review was not without limitations. We were unsuccessful in locating any recent case-control trials to include in our analysis regarding incidence (the last trial with suitable data for meta-analysis was conducted by Kuo et al. [35] in 2012), and furthermore, heterogeneity in the cohorts was extremely high (I^2^ = 98%). Additionally, several studies adjusted for a number of different factors, but there was no internal consistency, so the elimination of well-known confounders (including smoking) is impossible. Other information included dosage, compliance, treatment duration, and statin type. This was most often due to database limitations. Additionally, the population characteristics in the included studies were not uniform. An example of this is the trial report by Clearfield et al. [27] that only reported data for women. Finally, due to our keyword selection, it is likely that reports investigating statin effects that also had information on cancer incidence, but did not exclusively state it, were missed. We believe this number to be very small and with marginal capacity for impacting our results.

## 5. Conclusions

The current systematic review and meta-analysis focused on the effects of statins on UBC incidence and overall prognosis. Our results suggest no change in UBC risk between statin users and non-users. In addition, this review provides strong evidence of no association between statin use and UBC local control, recurrence, survival or mortality, and of the absence of any impact of statin use on the effectiveness of BCG immunotherapy. Our review revealed potential gaps in the literature that could be worth investigating in the future, such as the role of statins as adjunct chemotherapeutics in UBC. The anti-inflammatory and chemopreventive actions of statins have been documented in the past; however, these effects have not been investigated in targeted trials for UBC specifically.

## Figures and Tables

**Table 1:** Characteristics of the included studies.

First Author	Study Type	Country	Exposure and Comparator	Population (Exposure/Controls)	Outcome(s) Reported (Exposure/ Controls)	Characteristics and Comorbidities	Study Conclusion
Clearfield, M. et al., 2001 [27]	RCT	USA	Lovastatin vs. Placebo	499/498	Incidence 1/0	Women 55–73 years.	Incidence similar in both groups.
Graaf, M.R. et al., 2004 [32]	Case-Control	Netherlands	Any statin vs. No statin	1130/18,661	Incidence 249/986	Patients with prescriptions of cardiovascular drugs.	Suggested protective effect of statins against cancer.
Kaye, J.A. et al., 2004 [33]	Case-Control	UK	Any statin vs. No statin	3244/14,844	Incidence 19/74	Patients 50–89 years old who used antihyperlipidemic drugs or had a recorded diagnosis of untreated hyperlipidemia and age-matched controls.	Statin use does not have a substantial effect on cancer risk.
Strandberg, T.E. et al., 2004 [28]	RCT	Nordic countries ^§^	Simvastatin vs. Placebo	2221/2223	Incidence 19/17	CHD patients.	Incidence similar in both groups.
Heart Protection Study Collaborative Group, 2005 [29]	RCT	UK	Simvastatin vs. Placebo	10,269/10,267	Incidence 74/90	Patients 40–80 years with non-fasting blood total cholesterol concentrations of at least 135 mg/dL with history of occlusive arterial disease; diabetes mellitus; or treated hypertension.	No adverse effects of statins on cancer incidence.
Hoffmann, P. et al., 2006 [49]	Cohort	USA	Any statin vs. No statin	19/65	Progression 10/12 Cystectomy 8/9	Patients receiving BCG for NMIBC.	Discontinuation of statins during BCG therapy might be beneficial.
Sato, S. et al., 2006 [36]	Cohort	Japan	Pravastatin vs. No statin	179/84	Incidence 3/0	CHD patients aged 70 years or younger.	Significantly elevated risk of bladder cancer.
Tsai, H.K. et al., 2006 [43]	Cohort	USA	Any statin vs. No statin	35/251	Local control (UVA) 73%/52%	Patients with MIBC with maximal transurethral resection followed by chemoradiotherapy.	Statin use associated with improved LC on UVA but not after controlling for known prognostic factors.
Coogan, P.F. et al., 2007 [31]	Case-Control	USA	Any statin vs. No statin	190/3652	Incidence 20/216	Patients 40–79 years with a primary cancer of a site and regular users of statins.	No support for an association between statin use and cancer.
Kamat, A. et al., 2007 [44]	Cohort	USA	Any statin vs. No statin	39/117	Recurrence 23/69 Progression 12/33	Cohort of 156 patients receiving BCG immunotherapy.	No effect of statin use on recurrence, progression, or number of deaths during BCG therapy.
Rossebø, A.B. et al., 2008 [30]	RCT	USA	Simvastatin/Ezetimibe vs. Placebo	944/929	Incidence 7/7	Patients with mild-to-moderate asymptomatic aortic stenosis.	Similar incidence and risk between the two groups.
Berglund, R.K. et al., 2008 [45]	Cohort	USA	Any statin vs. No statin	Total 952, 245/707	Recurrence 214/582 Progression to surgery 78/287	Cohort of 952 patients treated with BCG immunotherapy.	No statistical difference in recurrence or progression to surgery.
Farwell, W.R. et al., 2008 [37]	Cohort	UK	Any statin vs. No statin	37,248/25,594	Incidence 326/258	Patients using antihypertensive medications but no cholesterol-lowering medications.	Incidence rate lower among statin users. Lower incidence during entire follow-up for users.
Friedman, G.D. et al., 2008 [38]	Cohort	USA	Any statin: any duration vs. ≥5 years	Total 353,199	Incidence 498 (418 men, 80 women)/111 (94 men, 17 women)	Statin users.	No strong evidence but observed increased risk for bladder cancer in both men and women.
Karp, I. et al., 2008 [39]	Cohort	Canada	High-dose statin vs. Low-dose statin vs. No statin ^†^	High-dose 6015 Low-dose 5323 None 18,738	Incidence (per group) 9/13/73	Patients aged ≥ 45 years discharged with a history of MI.	Suggested dose-response effect of lipophilic statins on cancer occurrence.
Skolarus, T.A. et al., 2009 [51]	Cohort	USA	Any statin vs. No statin	43/47	Progression 6/6 Mortality 3/2 Mortality 11/16	Patients diagnosed with UBC and treated with BCG immunotherapy.	No association of treatment outcomes with statin use.
Haukka, J. et al., 2010 [40]	Cohort	Finland	Any statin vs. No statin	472,481/472,781	Incidence 944/845	Individuals who purchased statins with no history of cancer.	Weak association between statin use and incidence.
Jacobs, E.J. et al., 2011 [41]	Cohort	USA	Any statin (subdivided into former use, current use < 5 y, current use > 5 y) vs. No statin	Former 5387/ Current (<5 y) 13,313/ Current (>5 y) 10,250/ No use 104,305	Incidence 1081/ Former 43/ Current (<5 y) 202/ Current (>5 y) 203/ No use 633	Participants of the CPS-II nutrition cohort.	Long-term use does not increase cancer risk.
Vinogradova, Y. et al., 2011 [34]	Case-Control	UK	Any statin (subdivided with treatment duration) vs. No statin	4227/17,559	Incidence 856/3125	Open cohort. Identified patients aged 30–100 with a history of cancer. Allocated 5 controls per case.	Non-significant increased risk observed.
Kuo, C.C. et al., 2012 [35]	Case-Control	Taiwan	Any statin vs. No statin	268/1032	Incidence 64/261	National health insurance (NHI). Patients aged ≥ 50 years, first-time diagnosed with UBC. Controls, patients with admission unrelated to statin use.	No association between statin use and UBC risk.
Segal, R. et al., 2012 [53]	Cohort	Canada	NA	NR/NR	Total 278 HR: 0.784 (95% CI 0.453–1.341, *p* = 0.375)	Cohort of 2570 patient records with T1HG UBC.	Statin use was not associated with worse prognosis.
Crivelli, J.J. et al., 2013 [46]	Cohort	USA	Any statin vs. No statin	341/776	NA	Patients with NMIBC treated with TURB.	Statin use was not associated with disease recurrence, progression, cancer-specific mortality, or any-cause mortality. Similar results in subgroup analyses.
da Silva, R.D. et al., 2013 [14]	Cohort	USA	Any statin vs. No statin	642/860	Disease recurrence (UVA, MVA) HR 1.22 (95% CI: 1.03–1.46, *p* = 0.02) HR 1.04 (95% CI: 0.86–1.24, *p* = 0.66) Ca specific mortality (UVA, MVA) HR 1.26 (95% CI: 1.04–1.54, *p* = 0.02) HR 1.04 (95% CI: 0.84–1.28, *p* = 0.68)	Patients treated with radical cystectomy and pelvic lymphadenectomy without neoadjuvant therapy.	Statin use was associated with disease recurrence and cancer specific mortality on UVA, but not on MVA.
Pastore, A.L. et al., 2015 [47]	Cohort	Italy	Statin (±Aspirin) vs. None or Aspirin only	189/385	Recurrence UVA OR 1.853 (95% CI: 1.144–3.1, *p* = 0.012) OR 1.886 (95% CI: 1.095–3.247, *p* = 0.022)	Patients with NMIBC treated with TURB.	Aspirin and statins are able to modify the behavior of NMIBC. Statins and combination treatment with aspirin groups showed increased recurrence rates and progression.
Richard, O.P. et al., 2017 [54]	Cohort	Canada	Any statin vs. No statin	4748/9063	CSS Before diagnosis: HR 1.04 (95% CI: 0.99–1.09, *p* = 0.43) After diagnosis: HR 1.04 (95% CI: 0.99–1.09, *p* = 0.10) OS Before diagnosis: HR 1.01 (95% CI: 0.99–1.03, *p* = 0.10) After diagnosis: HR 0.93 (95% CI: 0.91–0.96, *p* < 0.001)	Patients ≥ 66 years diagnosed with NMIBC with no record of statin use before that age.	Cumulative statin use was associated with an improvement in OS but not CSS.
Singla, N. et al., 2017 [50]	Cohort	USA	Any statin	64/35	Recurrence HR 0.93 (95% CI: 0.56–1.54, *p* = 0.764) Stage progression HR 0.72 (95% CI: 0.23–2.27, *p* = 0.574) Cystectomy HR 1.40 (95% CI: 0.58–3.37, *p* = 0.449) Overall mortality HR 0.76 (95% CI: 0.31–1.88, *p* = 0.554) Cancer-specific mortality HR 0.27 (95% CI: 0.05–1.49, *p* = 0.133)	Patients receiving intravesical BCG therapy for high-grade NMIBC.	No effect of statins on any of the oncologic outcomes.
Guercio, V. et al., 2019 [55]	Case-Control	Italy	Any statin vs. No statin	71/618 for cases 121/1233 for both groups	Total 690 71/0 *	UBC case-control study patients and hospital controls. Patients (cases) diagnosed with UBC.	Statin use does not increase cancer risk.
Lundberg, E. et al., 2019 [56]	Cohort	Sweden	Any statin vs. No statin	7754 of 22,936/68,247 of 229,326	Occurrence UBC OR 1.23 (95% CI: 1.19–1.27, *p* < 0.001) NMIBC OR 1.31 (95% CI: 1.26–1.35, *p* < 0.0001) MIBC OR 1.02 (95% CI: 0.96–1.08, *p* = 0.6)	Patients with diagnosed UBC and matched controls.	Statins were significantly associated with an increased risk of UBC.
Brooks, N.A. et al., 2021 [52]	Cohort	USA	Any statin vs. No statin	244/334 (1 unknown)	NA	Patients with NMIBC treated with BCG immunotherapy at least once.	No data for statins, but BMI (of ≥25 kg/m^2^) was significantly associated with improved PFS, OS, and CSS.
Ferro, M. et al., 2021 [48]	Cohort	Italy	Any statin vs. No statin	402/1108	MVA Recurrence HR 0.80 (95% CI: 0.67–0.95, *p* = 0.009) Progression HR 0.97 (95% CI: 0.79–1.19, *p* = 0.753) Overall mortality HR 0.71 (95% CI: 0.50–1.03, *p* = 0.068)	Patients with first diagnosis of T1 HG NMIBC after TURB.	Statin users exhibited lower disease rates, disease progression, and similar overall mortality compared to non-users. No adverse effect of statins on BCG efficacy.
Haimerl, L. et al., 2022 [57]	Cohort	Germany	Any statin vs. No statin	972	UVA RFS 174/685, *p* = 0.653 CSS 203/769, *p* = 0.296 OS 203/769, *p* = 0.482	Database of UBC patients who underwent radical cystectomy.	No correlation between statin use and RFS, CSS, or OS.
Halámková, J. et al., 2022 [42]	Cohort	Czech Republic	Any statin vs. No statin	53/304	Incidence 6/11	Adult patients with a histologically confirmed colorectal cancer diagnosis.	Use of hypolipidemic agents was associated with a lower incidence of an SPM, where the protective effect was most prominent in statin users.

^§^ Nordic countries include Denmark, Finland, Iceland, Norway, and Sweden. ^†^ Statins included: atorvastatin, simvastatin, lovastatin, fluvastatin. * Missing data. Abbreviations: BCG: bacille Calmette–Guérin, BMI: body mass index, CHD: coronary heart disease, CI: confidence interval, CSS: cancer-specific survival, HR: hazard ratio, LC: local control, MI: myocardial infarction, MIBC: muscle-invasive bladder cancer, MVA: multivariate analysis, NA: not available, NMIBC: non-muscle invasive bladder cancer, OS: overall survival, RCT: randomized controlled trial, RFS: recurrence-free survival, SPM: secondary primary malignancy, T1HG UBC: type-1 high-grade urinary bladder cancer, TURB: transurethral resection of the bladder, UBC: urinary bladder cancer, UK: United Kingdom, USA: United States of America, UVA: univariate analysis.

**Table 2:** Assessment of case-control studies using Newcastle–Ottawa scale.

Study	Selection	Comparability	Exposure	Total Score
Graaf, M.R. et al., 2004 [32]	★★★☆	★☆	★★☆	6
Kaye, J.A. et al., 2004 [33]	★★★★	★★	★★☆	8
Coogan, P.F. et al., 2007 [31]	★★★☆	★★	★★☆	7
Vinogradova, Y. et al., 2011 [34]	★★★★	★★	★★☆	8
Kuo, C.C. et al., 2012 [35]	★★★☆	★☆	★★☆	6
Guercio, V. et al., 2019 [55]	★★★☆	★★	★★☆	7
Mean				7

**Table 3:** Assessment of cohort studies using Newcastle–Ottawa scale.

Study	Selection	Comparability	Outcome	Total Score
Hoffmann, P. et al., 2006 [49]	★★☆☆	★☆	★★☆	5
Sato, S. et al., 2006 [36]	★★★☆	★★	★★★	8
Tsai, H.K. et al., 2006 [43]	★★★☆	★☆	★★★	6
Kamat, A. et al., 2007 [44]	★★★☆	★★	★★★	8
Berglund, R.K. et al., 2008 [45]	★★★★	★☆	★★★	8
Farwell, W.R. et al., 2008 [37]	★★★☆	★★	★★★	8
Friedman, G.D. et al., 2008 [38]	★★★★	★★	★★★	9
Karp, I. et al., 2008 [39]	★★★★	★☆	★★☆	7
Skolarus, T.A. et al., 2009 [51]	★★★☆	★★	★★★	8
Haukka, J. et al., 2010 [40]	★★☆☆	★☆	★★★	6
Jacobs, E.J. et al., 2011 [41]	★★☆☆	★★	★★★	7
Segal, R. et al., 2012 [53]	★★★★	★★	★★★	9
Crivelli, J.J. et al., 2013 [46]	★★★★	★★	★★☆	8
da Silva, R.D. et al., 2013 [14]	★★★☆	★★	★★★	8
Pastore, A.L. et al., 2015 [47]	★★★★	★★	★★★	9
Richard, O.P. et al., 2017 [54]	★★★☆	★☆	★★★	7
Singla, N. et al., 2017 [50]	★★★★	★★	★★★	9
Lundberg, E. et al., 2019 [56]	★★★★	★☆	★★★	8
Brooks, N.A. et al., 2021 [52]	★★★★	★★	★★★	9
Ferro, M. et al., 2021 [48]	★★★★	★☆	★★★	8
Haimerl, L. et al., 2022 [57]	★★★★	★★	★★★	9
Halámková, J. et al., 2022 [42]	★★★★	★☆	★★★	8
Mean				7.82

## Data Availability

Not applicable.

## Supplementary Materials

The following supporting information can be downloaded at: https://www.mdpi.com/article/10.3390/curroncol30070488/s1, Supplementary Table S1: PICOS criteria for inclusion and exclusion of studies; Supplementary Table S2: Excluded studies with reasons [12,13,14,15,16,17,18,19,20,21,22,23,24,25,26]; Supplementary Table S3: Studies included in meta-analysis [27,28,29,30,36,37,39,40,41,42].
