# Supplementary material for: The Effect of Statins on the Incidence and Prognosis of Bladder Cancer: A Systematic Review and Meta-Analysis
Source: Curr Oncol. 2023 Jul 12;30(7):6648–65. doi: 10.3390/curroncol30070488 (PMC10378493; doi:10.3390/curroncol30070488)
Supplement: Supplementary file 1 [file curroncol-30-00488-s001.zip › curroncol-2439015-supplementary.pdf]

The Effect of Statins on Incidence and Prognosis of Bladder Cancer: A Systematic Review and Meta-Analysis

Supplementary material

Supplementary Table S1. PICOS criteria for inclusion and exclusion of studies

Parameter	Inclusion Criteria	Exclusion Criteria
**Population**	All patients receiving statins (age >18 years)	Patients not receiving statins
**Intervention**	Administration of statins, alone or in conjunction with background treatment	Other interventions
**Comparator**	Placebo, controls, or any other intervention	Does not apply
**Outcome**	Effect on bladder cancer (incidence or prognosis)	Other outcomes or lack of data regarding effects of statins on bladder cancer.
**Study Design**	Randomized control trials, case-control, and cohort studies, published in English	Other study designs<br>Published in any other language than English

Supplementary Table S2. Excluded studies with reasons

First Author	Year	Reason of Exclusion
Ford I [12]	2007	UBC data not reported separately.
Tseng CH [13]	2011	No data for statin use.
Atmaca [14]	2014	Does not meet study design inclusion criteria.
Ozgur BC [15]	2014	Does not meet study design inclusion criteria.
Fujimoto M [16]	2015	No data for UBC.
Berger SV [17]	2016	No data for UBC.
Hu YB [18]	2018	No data for UBC.
Roy S [19]	2018	Statin use not reported. Insufficient data for UBC patients.
Giugliano RP [20]	2020	Comparator does not meet criteria.
Bedimo RJ [21]	2021	No data for UBC.
Okada S [22]	2021	No data for UBC.
Marrone MT [23]	2021	UBC data not reported separately.
Yeh JJ [24]	2021	UBC and kidney cancer data reported jointly. No distinct data for UBC reported.
Ahn J [25]	2022	UBC and kidney cancer data reported jointly. No distinct data for UBC reported.
Stepien K [26]	2022	No data for UBC.

Supplementary Table S3. Studies included in Meta-Analysis

First Author	Year	Study Design	Statin	Statin group (n)	Bladder cancer in statin group (n)	Control group (n)	Bladder cancer in control group (n)
Randomized Controlled Trials							
Clearfield M [27]	2001	RCT	Lovastatin vs Placebo	499	1	498	0
Strandberg TE[28]	2004	RCT	Simvastatin vs Placebo	2221	19	2223	17
Heart Protection Study Collaborative Group [29]	2005	RCT	Simvastatin vs Placebo	10269	74	10267	90
Rossebø AB [30]	2008	RCT	Simvastatin-Ezetimibe vs Placebo	943	7	929	7
Cohort Studies							
Sato S [36]	2006	Cohort	Pravastatin vs No statin	179	3	84	0
Farwell WR [37]	2008	Cohort	Any statin vs No statin	37248	326	25594	258
Karp I [39]	2008	Cohort	Any statin† vs No statin	11338	22	18738	73
Haukka J [40]	2010	Cohort	Any statin vs No statin	472481	944	472781	845
Jacobs EJ [41]	2011	Cohort	Any statin vs No statin	23563§	405§	104305	633
Halámková Jana [42]	2022	Cohort	Any statin vs No statin	52	6	304	11

List of studies included in the meta-analysis with extracted population data.
† Statins include atorvastatin, fluvastatin, lovastatin, simvastatin.
§ Population used included only current users.
